# Post-pandemic upsurge of group A streptococcus infections: potential link to impaired herd trained immunity following COVID-19 lockdowns

**DOI:** 10.3389/fimmu.2025.1684332

**Published:** 2025-10-15

**Authors:** Janusz Marcinkiewicz

**Affiliations:** Department of Infectious Diseases and Public Health, Faculty of Veterinary Medicine, University of Agriculture in Cracow, Krakow, Poland

**Keywords:** group A *streptococcus*, innate memory, herd trained immunity, pandemic lockdown, resurge of infections

## 1 Introduction

One of the most unexpected consequences of the COVID-19 pandemic was the resurgence of numerous bacterial and viral infections in its aftermath. Notably, the incidence of several infectious diseases in 2022–2023 returned to—or even surpassed—pre-pandemic levels (1–5). A number of high-income countries with well-developed healthcare systems, including the United Kingdom, Ireland, the Netherlands, France, Denmark, Sweden, the United States, Australia, and New Zealand, reported a marked increase in *Streptococcus pyogenes* (GAS) infections, particularly scarlet fever and invasive GAS hospitalizations, compared to pre-COVID-19 levels (6–8).

During the pandemic lockdown, widespread implementation of long-term non-pharmaceutical interventions (NPIs) led to a dramatic reduction in the incidence of various infectious diseases (9, 10). This was largely attributed to decreased circulation of environmental microbiota and pathogens, resulting in reduced natural exposure and a subsequent decline in population-level immunity (11). This concept—often referred to as “immunity debt”—is especially relevant to the post-pandemic resurgence of respiratory viral infections (e.g., RSV and influenza) and GAS infections (12–14). It underscores the relationship between reduced pathogen exposure and increased population susceptibility (15, 16). While several mechanisms have been proposed to explain this resurgence, a comprehensive explanation remains elusive.

A recent comprehensive review by Nygaard et al. in The Lancet explored possible contributors to the post-pandemic infection surge. Their analysis considered multiple factors, including antimicrobial resistance, reduced vaccine coverage, emergence of more virulent pathogen strains, virus–bacteria interactions, and altered immune function due to limited pathogen exposure. They concluded that the primary driver of the resurgence was the accumulation of immunity debt, coinciding with the re-emergence of common viral and bacterial pathogens (5, 10). Importantly, the role of COVID-19 in modulating trained immunity has been extensively investigated. However, to date, no association with upsurge of GAS infections has been reported (17–23).

It is challenging to formulate a universal explanation for this phenomenon, given the diversity of the pathogens involved—ranging from bacterial to viral, vaccine-preventable to non-vaccine-preventable, and those primarily governed by innate versus adaptive immunity. Nevertheless, the global lockdown created an unprecedented natural experiment in which an estimated four billion individuals experienced prolonged reduction in pathogen exposure (18). This scale of immunological isolation may never be replicated again, offering a rare opportunity to study population-level infectious immunity under such conditions (24, 25) In this study, we narrowed our focus to the post-pandemic surge in Group A *Streptococcus pyogenes* (GAS) infections. GAS lacks an available vaccine and is primarily controlled by innate immune mechanisms (26). Furthermore, this infection provides a unique lens through which it is possible to examine impaired trained (innate) immunity at the herd level—a phenomenon we previously described shortly after the COVID-19 pandemic (27).

### 1.1 *S. pyogenes* and the immune system: a model for post-pandemic immunity debt

Group A *Streptococcus pyogenes* (GAS) is a Gram-positive, beta-hemolytic bacterium that exclusively infects humans. It causes a range of diseases, most of which are self-limiting, but some can be severe or even fatal. GAS typically colonizes the upper respiratory tract and can evade host defense mechanisms, leading to conditions such as pharyngitis, pyoderma, scarlet fever, and invasive infections like necrotizing fasciitis and streptococcal toxic shock syndrome (STSS) (28–30).

GAS produces several virulence factors, including superantigens and the M1 protein. Superantigens, encoded by 13 distinct genes, induce massive T-cell proliferation and cytokine release, potentially triggering a cytokine storm and leading to sepsis (31). Such fatal outcomes are often linked to defects in the host’s innate immune response (15, 32). The M1 protein, another major virulence factor, prevents immunoglobulins from effectively attacking the pathogen by inhibiting immunoglobulin-mediated phagocytosis and enhancing resistance to neutrophil bactericidal activity (33). The M1 protein is part of the *emm* family, with over 250 distinct *emm* types identified based on variability in the N-terminal region. The *emm1* genotype, particularly the M1UK sub-lineage, first identified in the United Kingdom in 2019—has been associated with an increased incidence of scarlet fever and invasive GAS infections in the 21st century. Importantly, M1UK is strongly linked to an increase risk of STSS due to elevated superantigens production and enhanced ability to evade the immune system (14, 34). In addition, the M1UK lineage may acquire integrative conjugative elements (ICEs), thereby gaining new characteristics such as antibiotic resistance (26, 27, 35).

Notably, during the post-COVID-19 era, there has been a shift in the distribution of *emm* types in high-income countries, with *emm1* (especially M1UK) and *emm12* emerging as the most prevalent and virulent strains, capable of evading the immune system more effectively (36, 37).

### 1.2 Post-pandemic immunity debt and loss of trained immunity

Immunity debt, refers to the reduction in pathogen exposure and subsequent weakening of population immunity during the COVID-19 pandemic due to widespread non-pharmaceutical interventions (NPIs) (10). As a result, individuals experienced diminished levels of immunity typically acquired through regular exposure to pathogens. Proposed consequences of immunity debt include reduced antibody levels, a diminished pool of memory B and T cells, impaired innate defenses, and heightened susceptibility to infections. Recent reports have shown a decrease in circulating antibodies to common pathogens like respiratory syncytial virus (RSV) and GAS (13, 16). However, the impact of immunity debt on innate immunity, particularly on trained immunity, remains less well understood (38).

Trained immunity is a relatively new concept that redefines the role of the innate immune system in pathogen defense (39). Unlike the adaptive immune system, which develops antigen-specific memory, the innate immune system can “learn” from previous encounters with pathogens and enhance its response upon re-exposure. This process is triggered by interactions between microbiota and the host’s innate immune system (40). Notably, certain pathogens, vaccines (e.g., the BCG vaccine), and immune-modulating substances like β-glucans (derived from yeast species such as *Candida* and *Saccharomyces*) are potent inducers of trained immunity (41–43).

Trained immunity has revolutionized our understanding of innate immunity’s role in infectious disease defense. It enables a faster, more robust response to pathogens, preventing their multiplication and the onset of disease (44). The global reduction in microbial exposure during the COVID-19 lockdown has led to widespread depletion of trained immunity, weakening population-level defenses. As a result, herd trained immunity (HTI) has been impaired (27).

### 1.3 Innate immunity and GAS infections

The immune response to GAS infections is predominantly innate rather than adaptive. Recent studies have highlighted the role of macrophages and IFN-γ in protecting against GAS infection, with adaptive immune responses playing a lesser role in immediate defense (45). While adaptive immune responses can generate GAS-specific antibodies, these are often insufficient for long-term protection. Recurrent GAS infections do not confer effective, antigen-specific immunity, suggesting a critical role for the innate immune system in preventing infection. GAS exposure, however, does lead to the development of trained immunity (TI), wherein innate immune cells develop enhanced, non-specific memory and stronger responses to subsequent infections (32, 46, 47). Importantly, TI requires ongoing interaction with the microbiota, as the lifespan of trained immune cells, such as monocytes, is relatively short—typically no longer than three months (39, 44). In conclusion, the transmission and control of GAS infections may be governed more by population-level trained innate immunity rather than the generation of antigen-specific memory B and T cells (38–40). (41–44).

### 1.4 Herd immunity *vs*. herd trained immunity

The concept of herd immunity traditionally refers to the protection of a population from infectious disease through widespread acquisition of antigen-specific adaptive immunity, particularly by memory B and T cells (48). In contrast, herd trained immunity (HTI) refers to the defense mechanisms conferred by the innate immune system, which operates through nonspecific memory and enhanced responsiveness to pathogens. Unlike adaptive immune memory, trained immunity involves cells such as monocytes and macrophages that have a much shorter lifespan—typically weeks to months (40). I have created with the assistance of AI, the comparison table between classical herd immunity and my concept of HTI to clarify their impact on infectious immunity at the populational level (**Table 1**).

**Table 1:** Comparison of Classical Herd Immunity and Herd Trained Immunity (HTI).

Feature	Classical herd immunity	Herd trained immunity (HTI)
Underlying mechanism	Adaptive immune responses (pathogen-specific antibodies and memory T/B cells)	Innate immune system reprogramming (“trained immunity”) via epigenetic and metabolic changes
Specificity	Pathogen-specific, often strain-dependent	Broad, non-specific protection against diverse pathogens
Induction	Achieved through vaccination or natural infection	Achieved through repeated microbial exposure and environmental stimuli (e.g., commensal microbiota, vaccines with off-target effects)
Threshold concept	Requires a critical proportion of immune individuals to block transmission (R₀ dependent)	No fixed threshold; dependent on intensity and continuity of microbial stimulation across the population
Population effect	Reduces transmission of a specific pathogen, indirectly protecting non-immune individuals	Enhances baseline resistance to infections at the population level, potentially reducing severity and incidence of diverse pathogens
Durability	Long-lasting if immune memory is stable and pathogen evolution limited	Transient; may wane without sustained microbial exposure

## 2 Discussion

The COVID-19 pandemic lockdown created a unique and unexpected opportunity to investigate infectious immunity following a prolonged period of reduced exposure to common pathogens, including *Streptococcus pyogenes* (GAS). Of particular note is the sharp post-pandemic surge in GAS infections, with reported rates exceeding pre-pandemic prevalence by 2–4-fold (1, 2). This atypical rise is believed to be multifactorial, involving both host and pathogen-related factors, as postulated by previous studies.

Key contributing elements include: i) emergence or resurgence of virulent GAS strains, notably the *emm1* lineage (M1UK) (36). In Europe, early evidence indicated a predominance of the highly virulent M1UK variant; however, no corresponding increase in antibiotic resistance was observed (12, 49); ii) an expanded reservoir of asymptomatic GAS carriers, potentially increasing community transmission (8); iii) a rise in respiratory viral infections (e.g., RSV, influenza), which may predispose individuals to secondary bacterial infections, though these alone cannot fully explain the observed GAS increase (3); iv) immunity debt, a phenomenon characterized by reduced innate and adaptive immune preparedness due to decreased pathogen exposure during prolonged lockdown periods (38).

While each of these factors likely contributed to the unique epidemiological pattern observed, immunity debt—particularly its impact on trained innate immunity—appears to be the dominant driver. Trained immunity refers to a form of innate immune memory that enhances the host’s nonspecific defense mechanisms after repeated microbial encounters (31). At the population level, this concept extends to what has been termed Herd Trained Immunity (HTI), which complements classical antigen-specific herd immunity mediated by B and T cells. HTI may play a crucial role in preventing the spread of infections such as invasive GAS (iGAS) (27).

Importantly, the global pandemic restrictions have underscored the importance of HTI, a phenomenon that had not been widely recognized until now. While adaptive immune memory can persist for years, trained immunity offers a rapid, short-term defense that is crucial in controlling infections, especially in the absence of vaccine-induced immunity (45). The lockdown-induced reduction in pathogen exposure has thus exposed a critical vulnerability in our immune system—one that was previously underestimated. Impact of the long-term pandemic lockdown on overthrow of Herd Trained Immunity (HTI) is explained in **Table 2**.

**Table 2:** Impact of the long-term pandemic lockdown on overthrow of Herd Trained Immunity (HTI).

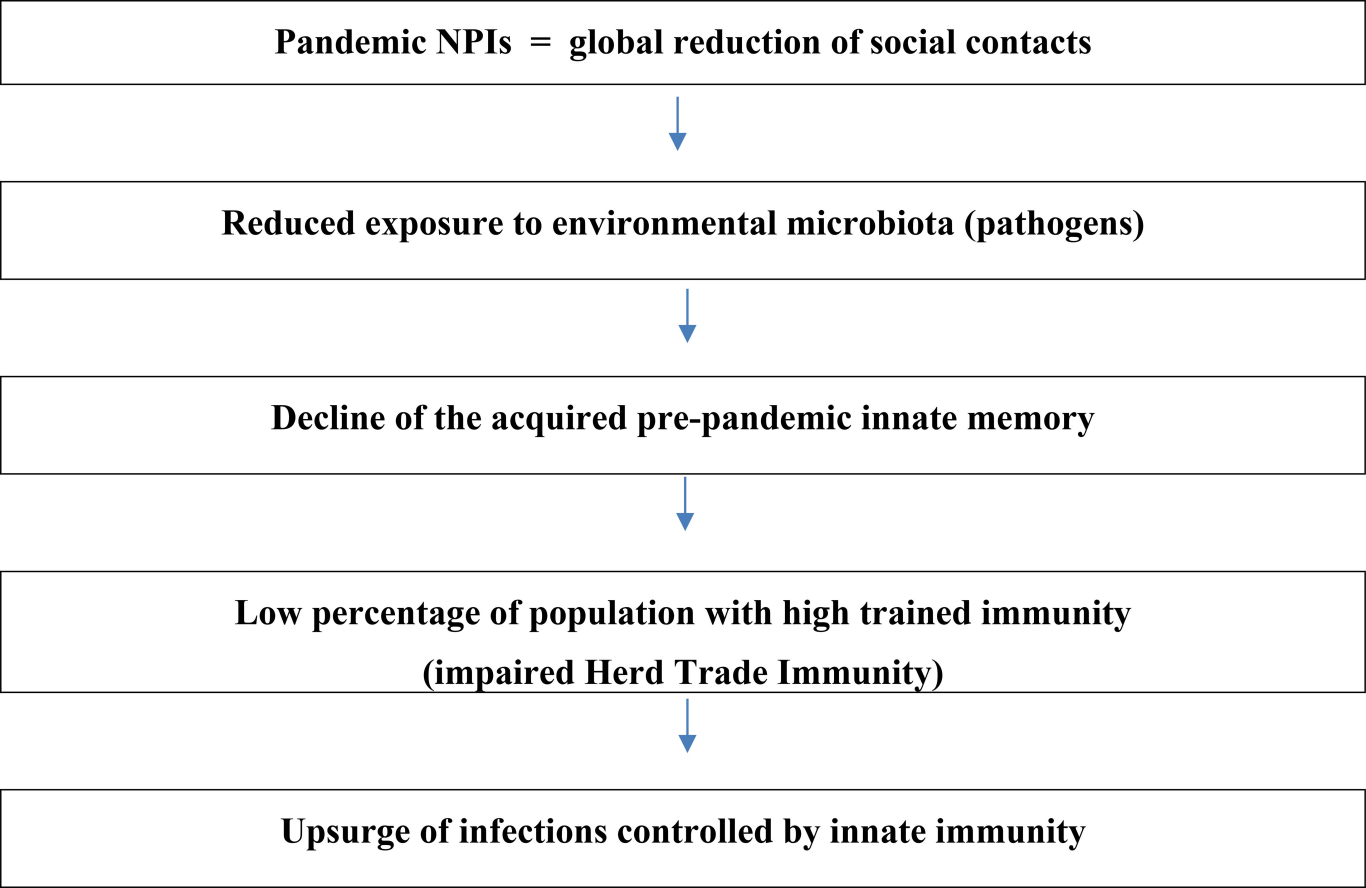

**Figure 1:**
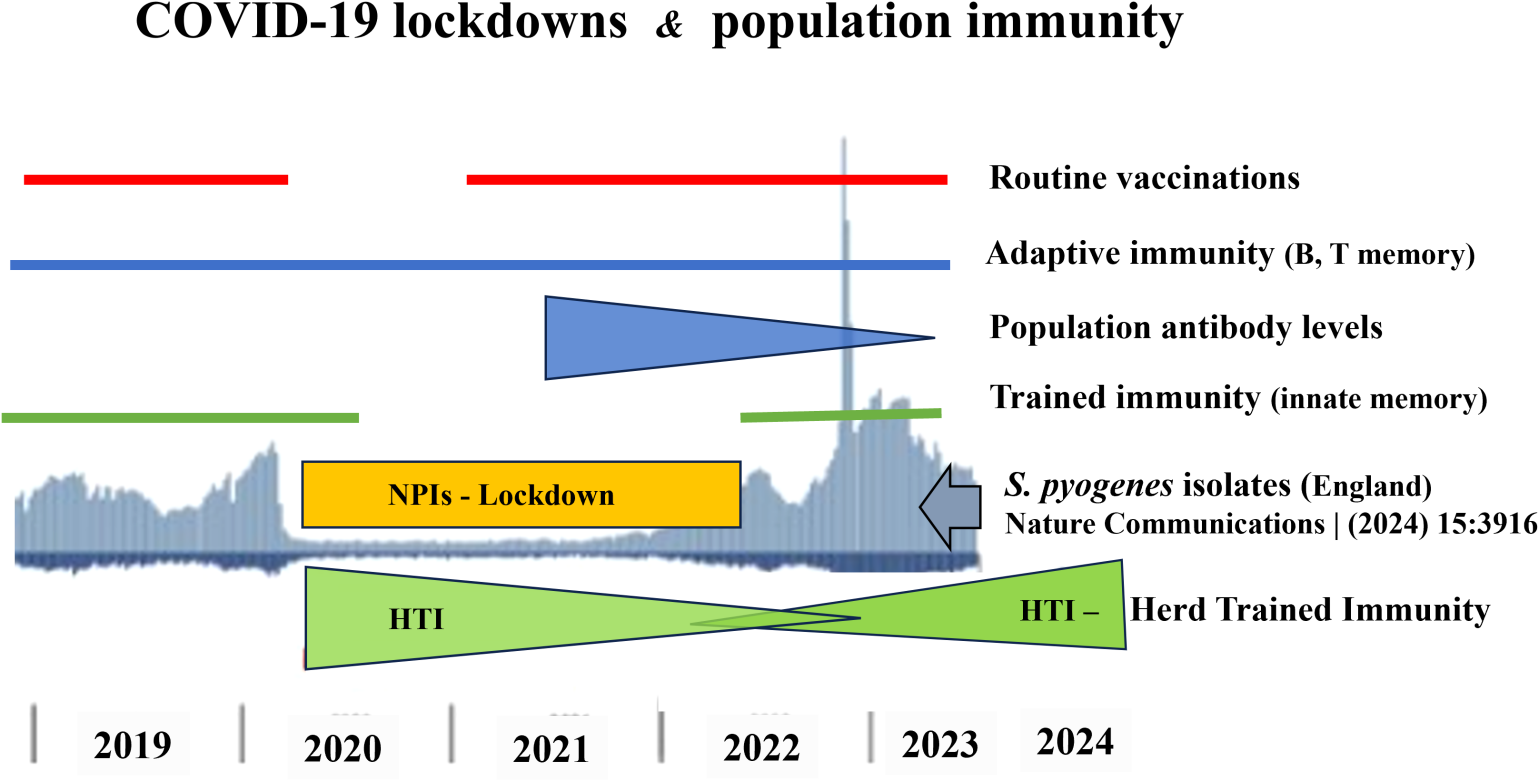
Timeline of *S. pyogenes* infections (2019–2024) and their relationship with the COVID-19 NPIs (reduced exposure to environmental microbiota 2020-2022), temporary suspension of routine vaccinations (2020), presence of pre-pandemic B and T memory cells (2019-2024), decline of population antibody levels (2021-2023), gap of trained immunity (2020-2022), disappearance (2020-2022) and re-creating of HTI (2022-2024).

Moreover, the post-pandemic resurgence of infections highlights the critical role of continual immune stimulation by environmental microbes in maintaining immune readiness. Social distancing and other NPIs, while essential in controlling SARS-CoV-2 transmission, inadvertently disrupted this natural immune training. Notably, trained immunity has a limited duration—estimated at around three months —suggesting that extended lockdowns led to the population-wide waning of innate immune protection acquired pre-pandemically (14, 23). The timeline of GAS infections in England (2019-2024), aligned with the implementation and relaxation of NPIs, further supports the hypothesis of impaired HTI during this period (**Figure 1**).

Crucially, the decline in GAS incidence observed in some countries during 2024–2025 provides compelling evidence for this hypothesis (49). This decline likely reflects the re-establishment of innate immune memory across populations through renewed microbial exposure, thereby restoring HTI (50). However, the pace of recovery of innate immunity varies between countries and depends on both the duration of lockdowns and the degree of compliance during the epidemic. In my opinion, the global incidence of GAS infections may return to pre-pandemic levels within two years. Nevertheless, strong evidence indicates that the incidence of extremely severe iGAS infections, such as STSS, has returned to pre-pandemic levels. For example, in Poland the reported number of STSS cases was 19–20 in the pre-pandemic years (2018-2019), decreased to 2 cases during the lockdown year (2020), surged to 100 cases in the post-pandemic period (2023), and declined to 15 cases in the current year (01.01-31.08.2025) (51, 52).

## 3 Conclusion

The COVID-19 pandemic lockdown has underscored the essential role of regular microbial exposure in sustaining immune competence. The post-pandemic surge in infections such as iGAS illustrates the impact of immunity debt—particularly innate immunity debt—resulting from prolonged NPIs. The emerging concept of HTI provides novel insights into the interplay between innate and adaptive immune responses and offers a new framework for understanding and managing infectious disease risks in the context of public health interventions. By acknowledging HTI, we can better inform future strategies for epidemic preparedness and immune system resilience.

Finally, this hypothesis, which associates impaired Herd Trained Immunity (HTI) with the post-pandemic resurgence of GAS infections, does not exclude the contribution of additional factors or triggers. Notably, it indicates for the first time, that population-level of trained innate immunity exists and may be compromised by prolonged reduced contact with microbiota. However, it should be confirmed in the future by retrospective epidemiological studies covering pre- and post-pandemic few years. Nevertheless, the observed association between COVID-19 lockdown and impaired HTI suggests that future pandemic response strategies should, in addition to widespread vaccination, carefully balance the extent of lockdown measures with the preservation of HTI.
